# SAMHD1 enhances HIV-1-induced apoptosis in monocytic cells via the mitochondrial pathway

**DOI:** 10.1128/mbio.00425-25

**Published:** 2025-05-28

**Authors:** Hua Yang, Pak-Hin Hinson Cheung, Li Wu

**Affiliations:** 1 Department of Microbiology and Immunology, Carver College of Medicine, The University of Iowa 311821 , Iowa City, Iowa, USA; University of California, Davis, California, USA

**Keywords:** SAMHD1, HIV-1 infection, apoptosis, THP-1 cells, monocytic cells, mitochondrial membrane potential, mitochondrial pathway, cytochrome c, BCL-2-interacting killer

## Abstract

Sterile alpha motif (SAM) and histidine-aspartate (HD) domain-containing protein 1 (SAMHD1) inhibits HIV-1 replication in non-dividing cells by reducing the intracellular dNTP pool. While SAMHD1 is known to promote spontaneous apoptosis, its role in HIV-1-induced apoptosis and the underlying mechanisms remain unclear. In this study, we identify a novel mechanism by which SAMHD1 enhances HIV-1-induced apoptosis in monocytic cells via the mitochondrial pathway. We demonstrate that SAMHD1 enhances apoptosis induced by HIV-1 infection in dividing monocytic THP-1 and U937 cell lines, but not in differentiated macrophage-like cells. Mechanistically, SAMHD1 expression reduces mitochondrial membrane potential and promotes cytochrome c release in HIV-1-infected THP-1 cells, thereby augmenting the mitochondrial apoptotic pathway. Furthermore, SAMHD1-enhanced apoptosis is linked to elevated levels of the pro-apoptotic protein BCL-2-interacting killer (BIK) in cells, which contributes to enhanced apoptosis during HIV-1 infection. These findings reveal a previously unrecognized regulatory role of SAMHD1 in amplifying HIV-1-induced apoptosis in monocytic cells, highlighting its involvement in the mitochondrial apoptotic pathway.

**IMPORTANCE:**

Sterile alpha motif (SAM) and histidine-aspartate (HD) domain-containing protein 1 (SAMHD1), a dNTP triphosphohydrolase, lowers intracellular dNTP levels and restricts HIV-1 replication in non-dividing cells. HIV-1 infection induces cell death mainly through apoptosis. While we have shown that endogenous SAMHD1 enhances spontaneous apoptosis in monocytic cells, its role in HIV-1-induced apoptosis and the underlying mechanisms remain unknown. In this study, we aim to bridge this knowledge gap by investigating the functional significance of SAMHD1 in regulating apoptosis during HIV-1 infection of immune cells. Our findings reveal a novel mechanism whereby SAMHD1 enhances HIV-1-induced apoptosis in monocytic cells through the mitochondrial pathway. This suggests a previously unrecognized role of SAMHD1 in modulating cellular responses to HIV-1 infection.

## INTRODUCTION

SAMHD1, a dNTP triphosphohydrolase, reduces intracellular dNTP levels (1, 2) and restricts the reverse transcription of retroviruses, including human immunodeficiency virus type 1 (HIV-1) (1, 3, 4) and certain simian immunodeficiency viruses (SIV) (5). Sterile alpha motif (SAM) and histidine-aspartate (HD) domain-containing protein 1 (SAMHD1) restricts HIV-1 infection in non-dividing immune cells, such as resting CD4+ T cells, macrophages, and dendritic cells (3, 4, 6). Through different mechanisms, SAMHD1 also restricts the replication of DNA viruses, including human papilloma virus (7), hepatitis B virus (8, 9), and human cytomegalovirus (10–12), as well as RNA viruses, including enteroviruses (13, 14) and influenza A virus (15, 16). We have previously shown that SAMHD1 suppresses NF-κB activation and type I interferon activation induced by virus infections and inflammation through interaction with the key proteins in their respective pathways (17–19). Multifaceted SAMHD1 also regulates a variety of cellular processes (20–22), including DNA replication fork progression (23), cell proliferation (7, 24), and apoptosis (24, 25). However, the mechanisms by which SAMHD1 regulates these processes remain unclear (20).

Apoptosis is a major form of programmed cell death and is divided into the death receptor apoptotic pathway and the mitochondrial apoptotic pathway (26, 27). In the death receptor apoptotic pathway, the binding of specific ligands, such as Fas ligand (Fas-L), to death receptors initiates the recruitment and activation of cysteine-aspartyl proteases (caspase) 8. Activated caspase 8 cleaves and activates downstream of caspases 3 and 7 (3/7) as executioners, which then cleave poly (ADP-ribose) polymerase (PARP), ultimately triggering apoptosis. In the mitochondrial apoptotic pathway, death receptor signals or mitochondrial apoptotic stimuli, including DNA damage and mitotic arrest, trigger mitochondrial outer membrane permeabilization (MOMP). This results in a decrease in the mitochondrial membrane potential (Δψm) and the release of cytochrome c (Cyto c) from the mitochondria into the cytosol. In the cytosol, Cyto c forms the apoptosome with apoptotic peptidase-activating factor 1, which activates the caspase cascade. Specifically, initiator caspase 9 is cleaved and activated, leading to the activation of caspases 3/7 to cleave a large set of substrates that ultimately lead to apoptotic cell death.

Specific substrates of caspases 3/7, such as cleavage of PARP (or PARP1), have been used as markers to indicate apoptosis. The mitochondrial apoptotic pathway is tightly regulated by the B-cell lymphoma 2 (BCL-2) protein family, which contains conserved BCL-2 homology (BH) domains (28). The BCL-2 protein family is divided into three subsets based on apoptotic function: anti-apoptotic BCL-2 proteins, including BCL-2, and B-cell lymphoma extra-large (BCL-XL); apoptotic effectors, including BCL-2-associated X (BAX) and BCL2 antagonist/killer (BAK); and pro-apoptotic BH3-only proteins, including BH3-interacting domain death agonist (BID), BCL-2-interacting mediator of cell death (BIM), and BCL2-interacting killer (BIK). The apoptotic effectors oligomerize and form pores in the outer mitochondrial membrane to induce MOMP. Anti-apoptotic BCL-2 proteins interact with apoptotic effectors to neutralize the change of MOMP, while BH3-only proteins interact with anti-apoptotic proteins BCL2, disrupting their inhibition of BAX/BAK, thereby promoting apoptosis (28).

HIV-1-infected individuals without effective antiretroviral therapy experience a progressive decrease in lymphocyte count, particularly CD4+ T cells (29). HIV-1 induces cell death primarily through apoptosis (30), pyroptosis (31, 32), necrosis, and necroptosis (33, 34). HIV-1 infection induces apoptosis through multiple viral proteins, including envelope glycoprotein (gp120), transcription activator (Tat), negative regulatory factor (Nef), and viral protein regulatory (Vpr) (35, 36). HIV-1 Vpr blocks the cell cycle at the G2 phase, which promotes apoptosis, and disrupts MOMP to facilitate the release of Cyto c (37, 38).

SAMHD1 restricts human T-cell leukemia virus type 1 (HTLV-1) infection of primary monocytes by accumulating viral reverse transcription intermediates, which activate the DNA sensing pathway and lead to apoptosis (39). We have demonstrated that exogenous SAMHD1 expression enhances Fas-L-induced apoptosis through the death receptor apoptotic pathway in T-cell lymphoma-derived HuT78 cells (40). We have also reported that endogenous SAMHD1 enhances spontaneous apoptosis in THP-1 cells (24). However, the function and mechanisms of SAMHD1 in regulating HIV-1-induced apoptosis remain unclear.

In this study, we found that endogenous SAMHD1 enhances apoptosis induced by HIV-1 infection in THP-1 cells through the mitochondrial pathway. We showed that SAMHD1 decreased Δψm and increased the release of Cyto c from the mitochondria into the cytosol in HIV-1-infected cells. Furthermore, SAMHD1-enhanced apoptosis was associated with increased BIK expression, and BIK contributed to SAMHD1-enhanced apoptosis during HIV-1 infection. Our findings reveal a regulatory mechanism by which SAMHD1 enhances apoptosis induced by HIV-1 infection, suggesting a previously unrecognized function of SAMHD1 in modulating cellular responses to viral infection.

## RESULTS

### Endogenous SAMHD1 enhances apoptosis induced by HIV-1 infection of THP-1 cells

**Fig 1:**
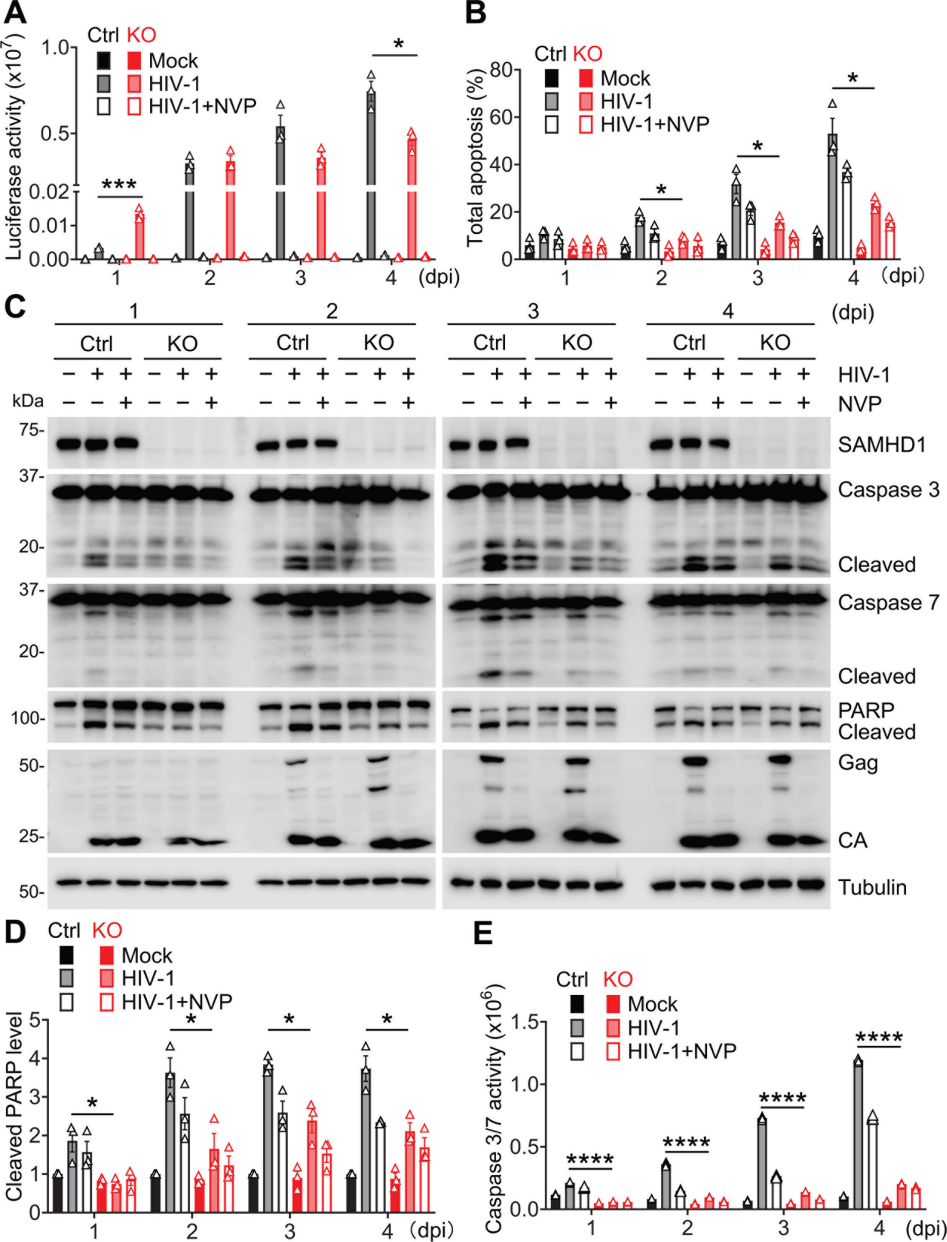
Endogenous SAMHD1 enhances apoptosis induced by HIV-1 infection of THP-1 cells. (**A through E**) THP-1 control (Ctrl) and stable SAMHD1 KO cell lines were infected with a single cycle, luciferase reporter HIV-1-Luc/VSV-G (multiplicity of infection [MOI] = 2) or mock infected. The HIV-1 reverse transcriptase inhibitor nevirapine (NVP) was used to treat infected cells to block HIV-1 infection. Cells were harvested at 1–4 days post-infection (dpi) for analysis. (**A**) HIV-1 infection levels were measured by luciferase assay and normalized to cellular protein concentration. (**B**) Apoptosis levels were measured by annexin V and 7-AAD staining and flow cytometry. The percentages of total apoptosis include early apoptosis (positive for annexin V but negative for 7-AAD) and late apoptosis (positive for both annexin V and 7-AAD). Original flow cytometry results are shown in Fig. S1A. (**C**) Detection of SAMHD1, caspases 3/7, poly(ADP-ribose) polymerase (PARP), HIV-1 Gag and capsids (CA), and tubulin by Western blot. Tubulin was used as a loading control. The cleaved caspases 3/7 and PARP proteins are indicated. (**D**) The cleaved PARP levels were quantified by densitometry analysis and normalized to tubulin. The level of cleaved PARP of mock-infected THP-1 Ctrl cells was set to 1. (**E**) Caspase 3/7 activities were measured by the Caspase-Glo 3/7 assay. (**A, B and D, E**) Data are presented as means ± SEM. The results in panels A, B, and D represent three independent experiments, and the results in panel E represent six independent experiments. The unpaired *t*-test was used for statistical significance compared with THP-1 Ctrl cells with HIV-1 infection. *P < 0.05; ***P < 0.001, ****P < 0.0001.

Our previous study demonstrated that SAMHD1 inhibited single-cycle HIV-1 infection in dividing THP-1 cells at 1 day post-infection (dpi), but not at 2 dpi (24). Similar HIV-1 infection levels at 2 dpi or later in dividing THP-1 cells with or without endogenous SAMHD1 provide an appropriate model to investigate the function of SAMHD1 in HIV-1-induced apoptosis in monocytic cells (24). To examine the effects of SAMHD1 on HIV-1 infection in the monocytic THP-1 cell line, THP-1 control (Ctrl) and SAMHD1 knock-out (KO) cell lines were infected with a single-cycle luciferase reporter HIV-1 (HIV-1-Luc/VSV-G), which lacks viral envelope protein and Nef (24). The HIV-1 reverse transcriptase inhibitor nevirapine (NVP) was used to treat infected cells to block HIV-1 replication. Luciferase activity, which indicates HIV-1 replication, was measured at 1–4 dpi. At 1 dpi, we detected higher luciferase activity in infected SAMHD1 KO cells compared to Ctrl cells, supporting that SAMHD1 inhibits HIV-1 replication at early time in THP-1 monocytic cells (Fig. 1A). At 2 and 3 dpi, however, luciferase activity became comparable between infected Ctrl cells and SAMHD1 KO cells. Conversely, at 4 dpi, higher luciferase activity was detected in infected Ctrl cells compared to SAMHD1 KO cells, consistent with our previous results that SAMHD1 did not inhibit HIV-1 infection at later time points in monocytic cells (24). As expected, NVP treatment completely inhibited HIV-1 replication from 1 to 4 dpi (Fig. 1A).

To analyze the effects of SAMHD1 on HIV-1-induced apoptosis in the monocytic cells, THP-1 Ctrl and SAMHD1 KO cell lines were infected with HIV-1-Luc/VSV-G for 1–4 days. Annexin-V and 7-AAD staining was used to detect early apoptosis (positive for annexin-V but negative for 7-AAD) and late apoptosis (positive for both annexin-V and 7-AAD). Flow cytometry analysis revealed that the percentages of total apoptosis were higher in infected THP-1 Ctrl cells compared to infected SAMHD1 KO cells at 2–4 dpi, suggesting that SAMHD1 enhances HIV-1-induced apoptosis in monocytic cells (Fig. 1B: Fig. S1A). NVP treatment partially inhibited HIV-1-induced apoptosis but did not affect spontaneous apoptosis (Fig. 1B; Fig. S1A and S2). As an example, our data showed that SAMHD1 expression increased total apoptosis by 2.1-fold in HIV-1-infected THP-1 cells at 3 dpi, whereas total apoptosis only increased by 1.6-fold in mock-treated THP-1 cells (Fig. 1B).

To investigate apoptosis occurring in productively infected or bystander cells, THP-1 Ctrl and SAMHD1 KO cell lines were infected with HIV-1-GFP/VSV-G or mock-infected at 3 dpi. NVP was used to block viral infection. The cells were stained with annexin V and 7-AAD and measured by flow cytometry. The infected cells (GFP positive) and bystander cells (GFP negative) were gated by the GFP channel. The percentages of GFP-positive cells in THP-1 Ctrl and SAMHD1 KO cell lines were 60% and 53%, respectively. NVP treatment completely blocked HIV-1-GFP expression. The flow cytometry results showed that both infected and bystander cells were induced for higher apoptosis by HIV-1 infection than uninfected cells (Fig. S3). SAMHD1 depletion reduced HIV-1-induced apoptosis in productively infected cells (1.79-fold decrease) and in bystander cells (3.68-fold decrease). SAMHD1 depletion also reduced spontaneous apoptosis (1.94-fold decrease). A stronger reduction in apoptosis was observed in bystander cells than that observed in infected cells and uninfected cells upon SAMHD1 depletion (Fig. S3). Therefore, endogenous SAMHD1 promotes HIV-1-induced apoptosis in both infected and bystander THP-1 cells.

To investigate the mechanisms by which SAMHD1 enhances apoptosis in cells, cleaved caspases 3/7 (effector caspases) and cleaved PARP (alternatively PARP-1, an apoptosis hallmark) were detected by Western blot. THP-1 Ctrl cells showed enhanced cleavage of caspases 3/7 and PARP upon HIV-1 infection compared to SAMHD1 KO cells at 1–4 dpi (Fig. 1C). The treatment of NVP partially inhibited HIV-1-induced cleavage of caspases 3/7 and PARP in both THP-1 Ctrl and SAMHD1 KO cells. Quantification of the cleavage of PARP revealed that HIV-1-induced PARP cleavage was stronger in THP-1 Ctrl cells than that in SAMHD1 KO cells at 1–4 dpi (Fig. 1D). Furthermore, HIV-1-Luc/VSV-G-infected THP-1 Ctrl cells showed higher caspase 3/7 activity than SAMHD1 KO cells at 1–4 dpi (Fig. 1E). NVP treatment partially inhibited HIV-1-induced caspase 3/7 activity. To investigate potential clonal effects, the HIV-1-Luc/VSV-G infection experiment was repeated in THP-1 Ctrl clone 2 and SAMHD1 KO clone 2 cells, which were different clones of THP-1 Ctrl and SAMHD1 KO cells, respectively. Consistent with clone 1, we found that HIV-1 induced better cleavage of caspase 3, caspase 7, and PARP in THP-1 Ctrl clone 2 than SAMHD1 KO clone 2 cells (Fig. S4). It was less likely that the clonal effect drove HIV-1-induced apoptosis in THP-1 Ctrl better than in SAMHD1 KO cells. Together, these results suggest that endogenous SAMHD1 enhances apoptosis induced by single-cycle HIV-1 infection in THP-1 cells.

### SAMHD1 reconstitution in THP-1 SAMHD1 KO cells enhances apoptosis induced by HIV-1 infection

**Fig 2:**
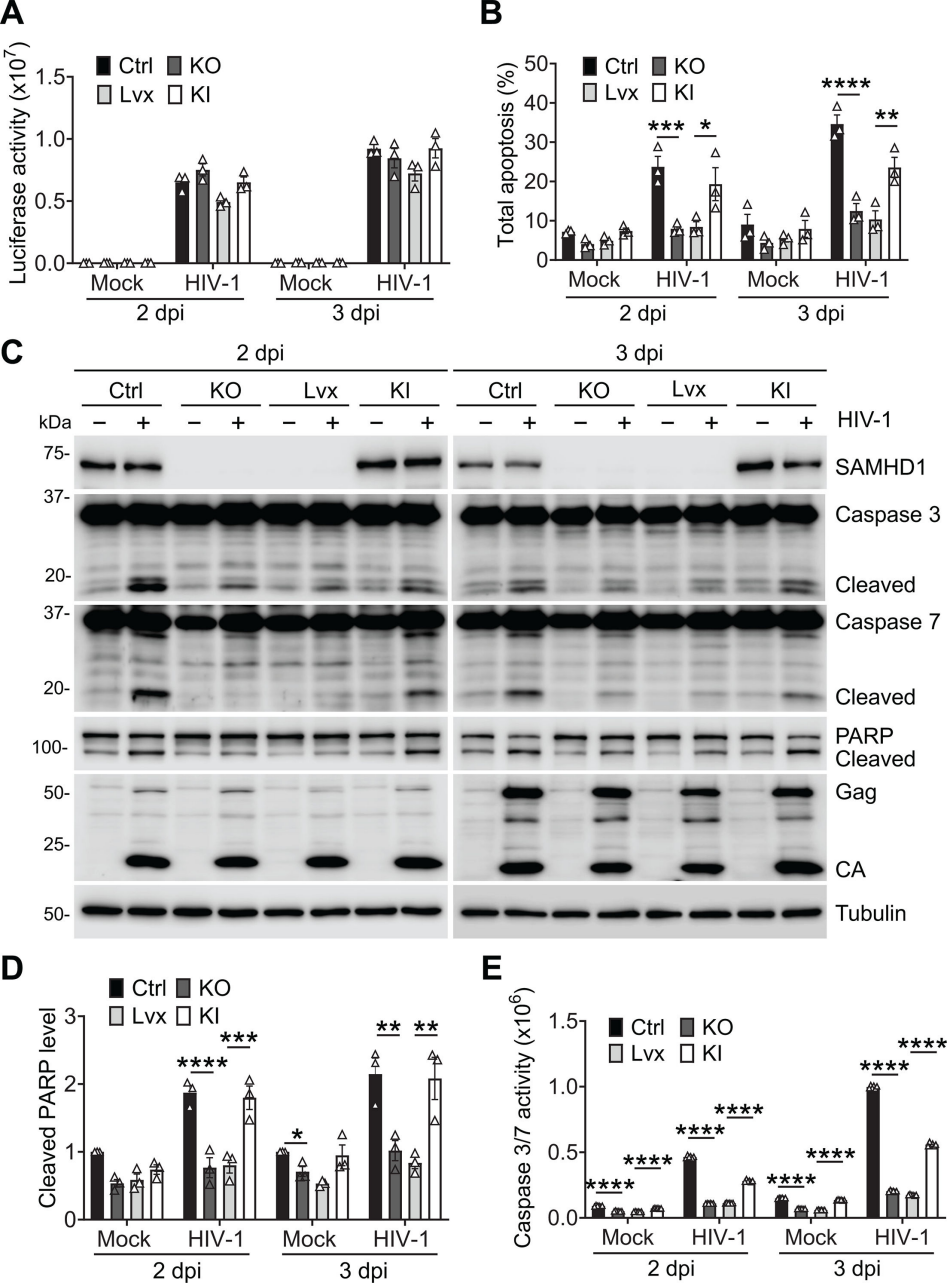
SAMHD1 reconstitution in THP-1 SAMHD1 KO cells enhances apoptosis induced by HIV-1 infection. (**A through E**) THP-1 Ctrl, SAMHD1 KO, Lvx vector control, and SAMHD1 KI cell lines were infected with HIV-1-Luc/VSV-G (MOI = 2) or mock treated. Cells were collected at 2 and 3 dpi for analysis. The data in panels A, B, and D (means ± SEM) represent three independent experiments, and the data in panel E represent six independent experiments. (**A**) The HIV-1 infection levels were measured by luciferase assay and normalized to cellular protein concentration. (**B**) Total apoptosis of cells was measured by flow cytometry as indicated in Fig. 1B. Original flow cytometry results are shown in Fig. S1B. (**C**) Detection of SAMHD1, caspase 3, caspase 7, PARP, HIV-1 Gag and CA, and tubulin by Western blot. The cleaved caspases 3/7 and PARP proteins are indicated. (**D**) The relative cleaved PARP levels were quantified by densitometry analysis and normalized to tubulin. The level of cleaved PARP of mock-infected THP-1 Ctrl cells was set to 1. (**E**) Caspase 3/7 activities were measured by Caspase-Glo 3/7 assay. (**A, B and D, E**) One-way ANOVA was used for statistical significance compared with THP-1 Ctrl cells or THP-1 Lvx cells. *P < 0.05; **P < 0.01; ***P < 0.001, ****P < 0.0001.

To confirm the function of SAMHD1 in enhancing apoptosis, we reconstituted SAMHD1 expression in THP-1 SAMHD1 KO cells and assessed HIV-1-induced apoptosis. THP-1 Ctrl, SAMHD1 KO, Lvx vector control, and SAMHD1 knock-in (KI) cell lines were infected with HIV-1-Luc/VSV-G or mock infected for 2 and 3 dpi. THP-1 Lvx vector control cells and SAMHD1 KI cells were generated from the parental SAMHD1 KO cells (24). HIV-1-Luc/VSV-G infection efficiency was similar across the four cell lines at 2 dpi and 3 dpi (Fig. 2A). The percentages of total apoptosis in THP-1 Ctrl and SAMHD1 KI cells, which expressed similar levels of SAMHD1 protein, were higher than that observed in SAMHD1 KO and Lvx cells upon HIV-1-Luc/VSV-G infection (Fig. 2B and C; Fig. S1B). THP-1 Ctrl and SAMHD1 KI cells exhibited enhanced cleavage of caspases 3/7 and PARP compared to SAMHD1 KO and Lvx cells upon HIV-1-Luc/VSV-G infection (Fig. 2C). Quantification of three independent experiments showed that the cleavage of PARP induced by HIV-1 infection was stronger in THP-1 Ctrl and SAMHD1 KI cells than SAMHD1 KO and Lvx cells at 2 and 3 dpi (Fig. 2D). Consistently, THP-1 Ctrl and SAMHD1 KI cells had higher HIV-1-induced caspase 3/7 activity compared to that observed in SAMHD1 KO and Lvx cells (Fig. 2E). Thus, SAMHD1 reconstitution in THP-1 SAMHD1 KO cells enhances apoptosis induced by single-cycle HIV-1 infection.

### NVP or Z-DEVD-FMK treatment inhibits SAMHD1-enhanced apoptosis induced by replication-competent HIV-1 infection

**Fig 3:**
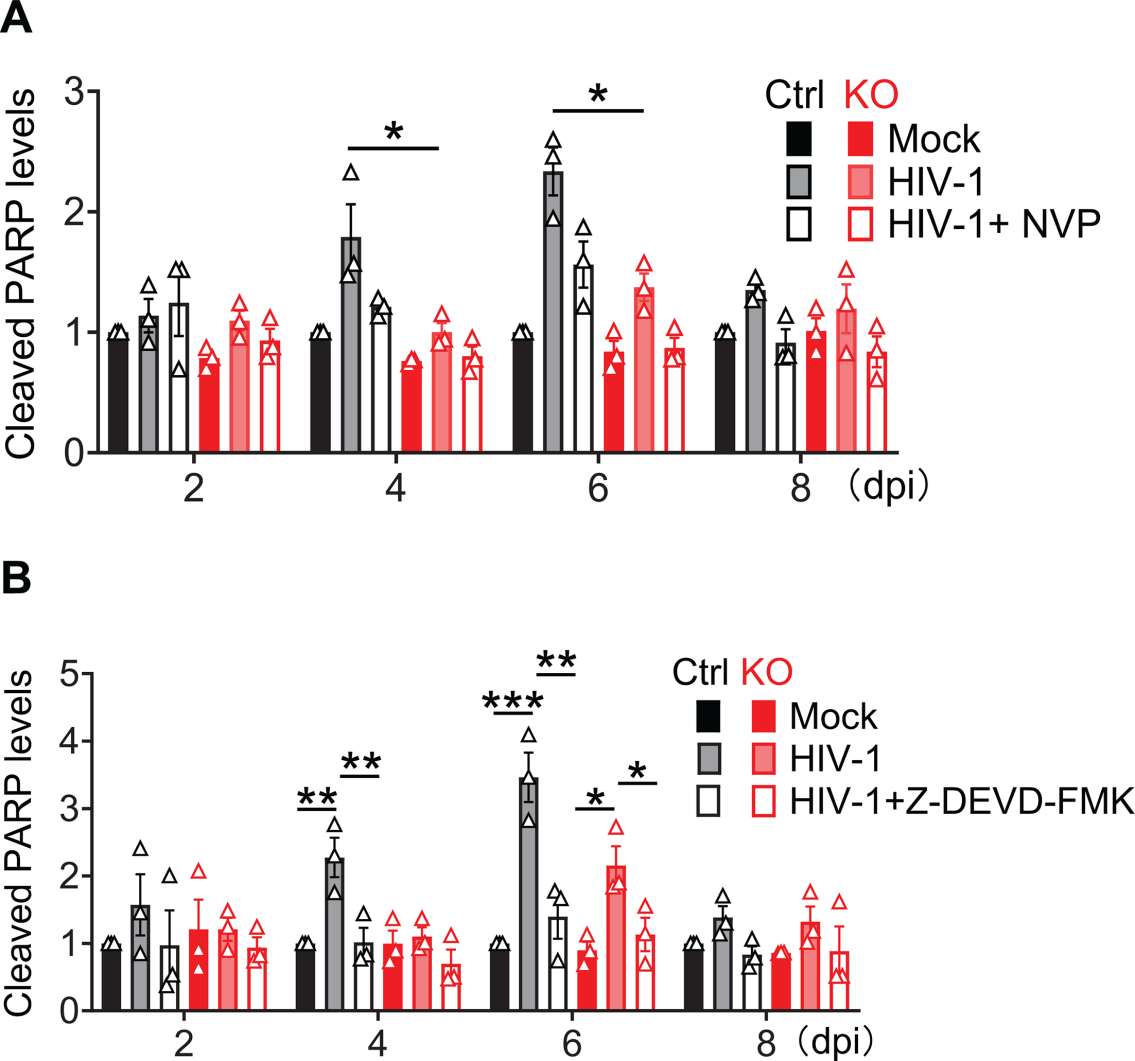
NVP or Z-DEVD-FMK treatment inhibits SAMHD1-enhanced apoptosis induced by replication-competent HIV-1 infection. (**A and B**) THP-1 Ctrl and SAMHD1 KO cell lines were infected with replication-competent HIV-1_NL4-3_ (MOI = 2) or mock treated and harvested at 2, 4, 6, and 8 dpi for cleaved PARP analysis by Western blot. The relative cleaved PARP levels were quantified by densitometry analysis, normalized to tubulin, and represented by three independent experiments. The level of cleaved PARP of mock-infected THP-1 Ctrl cells was set to 1. Unpaired t-test (**A**) and one-way ANOVA (**B**) were used for statistical significance compared with THP-1 Ctrl cells and SAMHD1 KO cells with HIV-1_NL4-3_ infection. *P < 0.05; **P < 0.01, ***P < 0.001. (**A**) NVP was used to block HIV-1 infection. Original Western blots are shown in Fig. S5A. (**B**) Z-DEVD-FMK was used to inhibit caspase 3/7 activity. Original Western blots are shown in Fig. S5B.

To investigate the role of SAMHD1 in apoptosis induced by wild-type replication-competent HIV-1 that expresses all viral proteins, THP-1 Ctrl and SAMHD1 KO cell lines were mock infected or infected with HIV-1_NL4-3_ in the presence or absence of NVP at 2, 4, 6, and 8 dpi. HIV-1_NL4-3_-infected THP-1 Ctrl cells generally exhibited a higher apoptotic response than the infected SAMHD1 KO cells at 4 and 6 dpi, as shown by quantification of the cleaved PARP levels (Fig. 3A). Compared to SAMHD1 KO cells, HIV-1_NL4-3_ infection in THP-1 Ctrl cells induced higher cleavage of caspases 3/7 and PARP at 4 and 6 dpi (Fig. 3A; Fig. S5A). Expression of HIV-1_NL4-3_ Gag (p55), immature Gag (p41), and capsid (CA) was similar between HIV-1-infected THP-1 Ctrl cells and SAMHD1 KO cells (Fig. S5A). NVP treatment inhibited HIV-1_NL4-3_ replication and HIV-1-induced apoptosis in both THP-1 Ctrl and SAMHD1 KO cells (Fig. 3A; Fig. S5A). Importantly, NVP treatment blocked HIV-1_NL4-3_-induced PARP cleavage in THP-1 Ctrl cells at 4 and 6 dpi (Fig. 3A). Therefore, SAMHD1 promotes apoptosis induced by replication-competent HIV-1 infection in dividing THP-1 cells.

To examine whether SAMHD1-enhanced apoptosis by HIV-1 infection is dependent on caspase 3/7 activity, THP-1 Ctrl and SAMHD1 KO cell lines were infected or mock infected with HIV-1_NL4-3_ and treated with Z-DEVD-FMK, which is a specific and irreversible caspase-3/7 inhibitor and widely used for inhibiting cell apoptotic death (41, 42). Z-DEVD-FMK treatment did not affect the expression of HIV-1 Gag, immature Gag, and CA, which were comparable between THP-1 Ctrl cells and SAMHD1 KO cells (Fig. S5B), suggesting similar HIV-1_NL4-3_ replication efficiency in these cells. Z-DEVD-FMK treatment significantly mitigated HIV-1_NL4-3_-induced PARP cleavage in THP-1 Ctrl cells at 4 and 6 dpi (Fig. 3B; Fig. S5B). HIV-1_NL4-3_ infection did not significantly induce PARP cleavage in SAMHD1 KO cells at 2, 4, and 8 dpi. Z-DEVD-FMK treatment also decreased HIV-1-induced cleavage of caspases 3/7 and PARP in THP-1 Ctrl at 4 and 6 dpi and in SAMHD1 KO cells at 6 dpi (Fig. 3B; Fig. S5B). These data suggest that caspase 3/7 activities contribute to SAMHD1-enhanced apoptosis induced by HIV-1 replication in dividing THP-1 cells.

### SAMHD1 enhances apoptosis induced by HIV-1 infection in monocytic cells but not in macrophage-like cells

**Fig 4:**
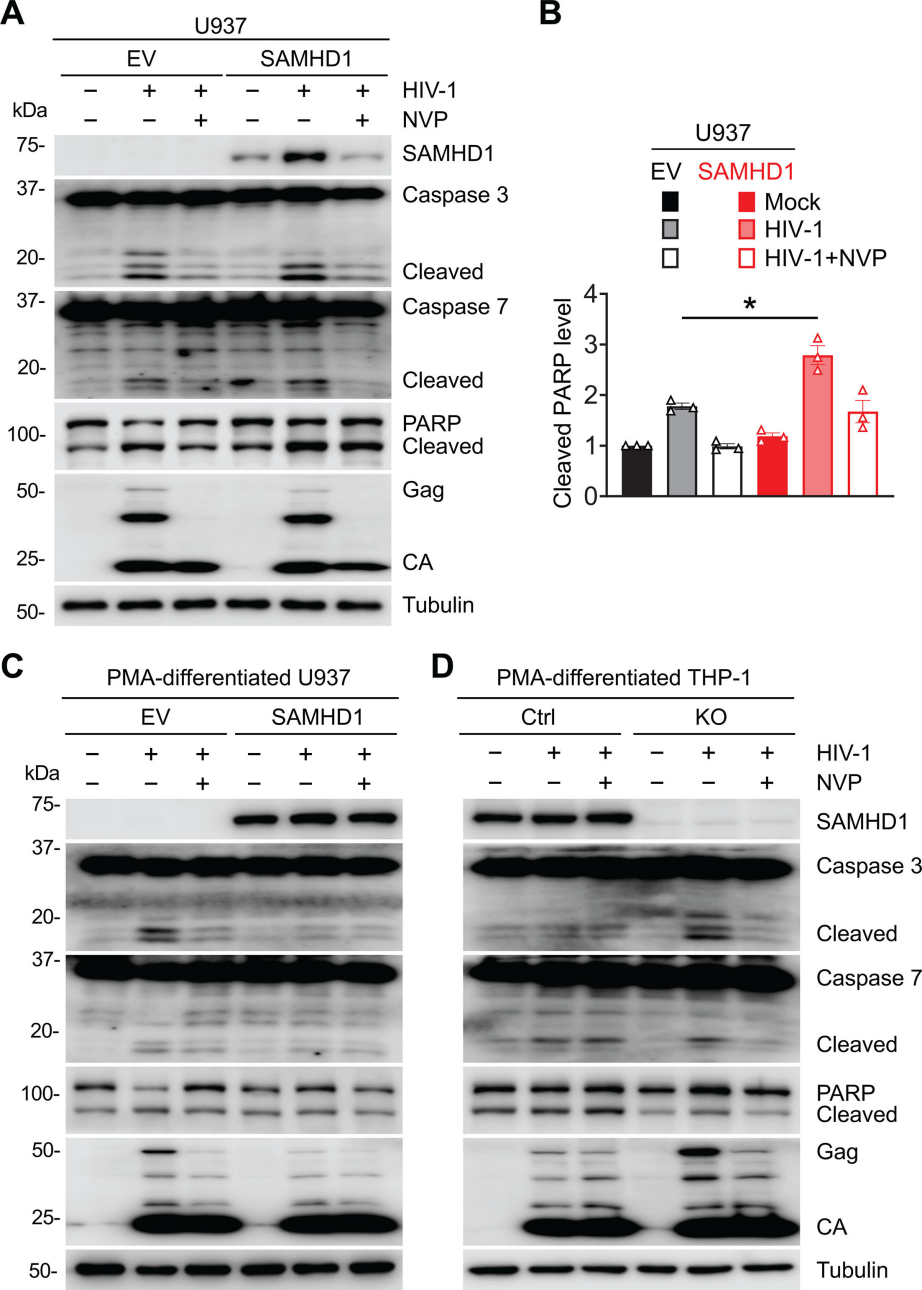
SAMHD1 enhances apoptosis induced by HIV-1 infection only in monocyte cells, not in macrophage-like cells. (**A and B**) U937 empty vector (EV) and SAMHD1 expression cell lines were infected with HIV-1-Luc/VSV-G (MOI = 2) or mock treated at 3 dpi. (**C and D**) PMA-differentiated U937 EV and SAMHD1 expression cell lines (**C**) and PMA-differentiated THP-1 Ctrl and SAMHD1 KO cell lines (**D**) were infected with HIV-1-Luc/VSV-G (MOI = 2) or mock treated at 3 dpi. NVP was used to treat infected cells to block HIV-1 infection. (**A, C, and D**) Detection of SAMHD1, caspases 3/7, PARP, HIV-1 Gag, HIV-1 CA, and tubulin by Western blot. Tubulin was used as a loading control. The cleaved caspases 3/7 and PARP proteins are indicated. (**B**) The relative cleaved PARP levels were quantified by densitometry analysis and normalized to tubulin. The level of cleaved PARP of mock-infected U937 EV cell lines was set to 1. The data (means ± SEM) represent three independent experiments. Unpaired t-test was used for statistical significance compared with U937 EV cells with HIV-1 infection. *P < 0.05.

To further validate the effects of SAMHD1 on HIV-1-induced apoptosis in the monocytic cells, another monocytic cell line U937, which expresses a very low level of SAMHD1, was used. U937 cells were transduced with a lentiviral vector and puromycin selected for stable expression of human SAMHD1 (U937 SAMHD1 cells) driven by CMV promoter as previously described (43). U937 cells transduced with empty lentiviral vector with CMV promoter (U937 EV cells) were used as a negative control. U937 EV cells and U937 SAMHD1 cells were infected with HIV-1-Luc/VSV-G for 3 days. HIV-1 infection levels in U937 EV cells and U937 SAMHD1 cells were similar, as indicated by comparable expression of Gag and CA protein (Fig. 4A). Interestingly, HIV-1 infection promoted SAMHD1 expression in U937 SAMHD1 cells, which was reverted by NVP treatment. SAMHD1 was undetectable in U937 EV cells both in the presence and absence of HIV-1 infection. Importantly, U937 SAMHD1 cells showed enhanced cleavage of caspases 3/7 and PARP upon HIV-1 infection compared to U937 EV cells (Fig. 4A). The treatment of NVP partially inhibited HIV-1-induced cleavage of caspases 3/7 and PARP in both U937 EV cells and U937 SAMHD1 cells. Quantification of the cleavage of PARP revealed that HIV-1-induced PARP cleavage was stronger in U937 SAMHD1 cells than in U937 EV cells (Fig. 4B). Thus, these data further validate the observation in THP-1 cells that SAMHD1 enhances apoptosis induced by HIV-1 infection in monocytic cells (Fig. 1 to 4A and B).

To investigate the role of SAMHD1 in HIV-1-induced apoptosis in macrophage-like cells, phorbol 12-myristate 13-acetate (PMA)-differentiated U937 and PMA-differentiated THP-1 were infected with HIV-1-Luc/VSV-G for 3 days. Unlike dividing monocytic cells, SAMHD1 is a potent restriction factor against HIV-1 replication in terminally differentiated macrophages (3, 4, 6). Indeed, SAMHD1 reconstitution suppressed HIV-1 infection in PMA-differentiated U937 cells as indicated by a decrease in expression level of Gag and CA (Fig. 4C). Similarly, SAMHD1 depletion promoted HIV-1 infection levels in PMA-differentiated THP-1 cells (Fig. 4D). Moreover, NVP treatment did not further suppress Gag and CA expression when SAMHD1 was sufficiently expressed (Fig. 4C and D). Therefore, SAMHD1 expression efficiently inhibited HIV-1 replication in the macrophage-like cells, which can no longer be suppressed by the reverse transcription inhibitor NVP.

We next decided to examine whether SAMHD1 still promoted HIV-1-induced apoptosis in the macrophage-like cells when HIV-1 replication was efficiently restricted. Apoptosis-related cleavage of PARP, caspase 3, and caspase 7 induced by HIV-1 was tested in PMA-differentiated U937 and PMA-differentiated THP-1 by western blot. As shown in Fig. 4C, SAMHD1 reconstitution suppressed HIV-1-induced cleavage of caspase 3 and 7 in PMA-differentiated U937 cells, like that observed in NVP treatment in U937 EV cells (Fig. 4C). Similarly, SAMHD1 depletion promoted HIV-1-induced cleavage of caspase 3 and 7 in PMA-differentiated THP-1 cells, which was reverted by NVP treatment (Fig. 4D). HIV-1 infection did not efficiently promote PARP cleavage in both PMA-differentiated U937 and PMA-differentiated THP-1 cells in the presence or absence of SAMHD1 expression compared with that observed in undifferentiated U937 and THP-1 cells (Fig. 4C and D vs Fig. 1 to 4A and B). Collectively, the results indicate that efficient restriction of HIV-1 replication by SAMHD1 in macrophage-like cells downregulates SAMHD1-enhanced apoptosis by HIV-1 infection. HIV-1 replication is required for SAMHD1-enhanced apoptosis to HIV-1 infection. SAMHD1 restricts HIV-1 replication in terminally differentiated macrophage-like cells but not in dividing monocytic cells. Therefore, SAMHD1 enhances HIV-1-induced apoptosis in monocyte cells but not in macrophage-like cells.

### SAMHD1 enhances apoptosis induced by HIV-1 infection through the mitochondrial pathway

To investigate whether SAMHD1 enhances apoptosis induced by HIV-1 infection through the mitochondrial apoptotic pathway, we measured Δψm of cells during HIV-1 infection. THP-1 Ctrl and SAMHD1 KO cell lines were mock infected or infected with single-cycle HIV-1-Luc/VSV-G and treated with NVP. At 1, 2, 3, and 4 dpi, cells were stained with JC-1 to detect Δψm using flow cytometry. JC-1 is a fluorescent dye that serves as an indicator of Δψm during apoptosis (44). When cells have high Δψm, JC-1 forms J-aggregates in mitochondria and exhibits red fluorescence. However, if Δψm decreases due to the damage of the mitochondrial membrane, JC-1 leaks into the cytoplasm and shifts to J-monomers, which emit green fluorescence. Therefore, the red/green ratio of the cell population with JC-1 staining correlates with Δψm and represents mitochondrial membrane integrity (44).

**Fig 5:**
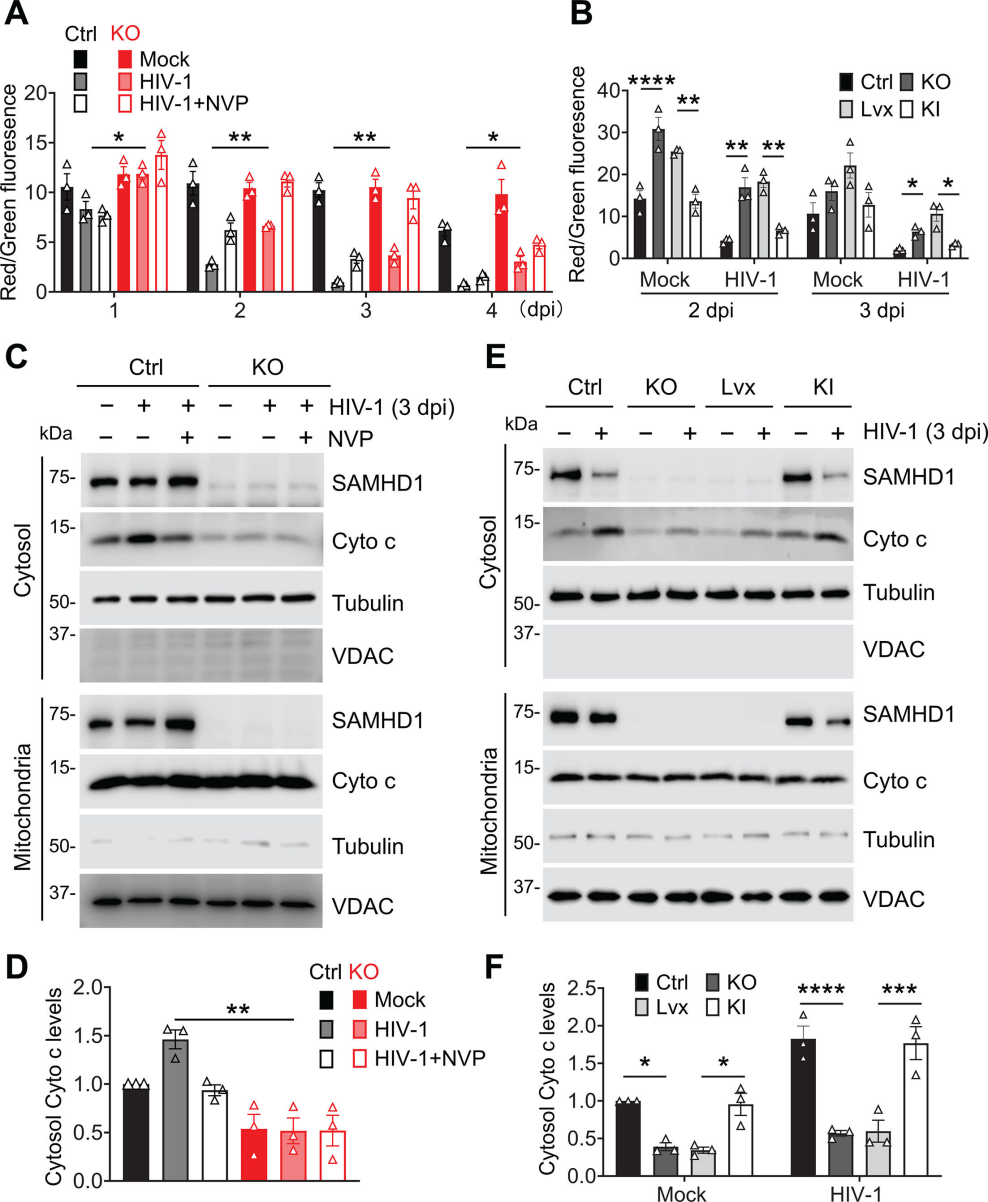
SAMHD1 enhances apoptosis induced by HIV-1 infection through the mitochondrial pathway. (A, C, and D) THP-1 Ctrl and SAMHD1 KO cell lines were infected with HIV-1-Luc/VSV-G (MOI = 2) or mock treated for the indicated times. NVP was used to block HIV-1 infection. (B, E, and F) THP-1 Ctrl, SAMHD1 KO, Lvx control, and SAMHD1 KI cell lines were infected with HIV-1-Luc/VSV-G (MOI = 2) for the indicated times. (**A and B**) Cells were stained with JC-1 and measured by flow cytometry. Original flow cytometry results are shown in Fig. S6A and B, respectively. (**C and E**) Detection of SAMHD1, cytochrome c (Cyto c), VDAC, and tubulin in the cytosol and mitochondrial fractions at 3 dpi by western blot. VDAC and tubulin were used as mitochondrial and cytosolic markers, respectively. (**D and F**) The relative levels of Cyto c in the cytosol fraction at 3 dpi (C and E, respectively) were quantified by densitometry analysis and normalized to tubulin. The level of Cyto c in the cytosol fraction of mock-infected THP-1 Ctrl cells was set to 1. (A, B, D, and F) The data (means ± SEM) represent three independent experiments. Unpaired t-test (**A and D**) and one-way ANOVA (**B and F**) were used for statistical significance. *P < 0.05; **P < 0.01, ***P < 0.001, ****P < 0.0001.

Interestingly, SAMHD1 expression decreased Δψm in the absence or presence of HIV-1-Luc/VSV-G infection, although infection potentiated the reduction (Fig. 5A; Fig. S6A). At 2, 3, and 4 dpi, HIV-1-Luc/VSV-G infection significantly decreased Δψm of THP-1 Ctrl cells, while the reduction in SAMHD1 KO cells was milder compared with that in THP-1 Ctrl cells. NVP treatment partially reverted Δψm reduction in both infected THP-1 Ctrl cells and SAMHD1 KO cells despite complete inhibition of HIV-1-Luc/VSV-G replication (Fig. 1A and 5A). This was consistent with the observation of partial inhibition of HIV-1-Luc/VSV-G induced apoptosis by NVP treatment (Fig. 1). Furthermore, SAMHD1 expression was reconstituted in SAMHD1 KO cells to confirm the role of SAMHD1 in mitochondrial depolarization with or without HIV-1-Luc/VSV-G infection. At 2 and 3 dpi, THP-1 Ctrl and SAMHD1 KI cells showed significantly less Δψm, which was further decreased by HIV-1 infection, compared to SAMHD1 KO and Lvx cells (Fig. 5B; Fig. S6B). For example, Δψm decreased 10-fold due to HIV-1 infection in THP-1 Ctrl cells at 3 dpi, whereas in THP-1 SAMHD1 KO cells, HIV-1 infection reduced Δψm by 2.8-fold (Fig. 5A and B). These results indicated that SAMHD1 depolarized mitochondria, which were enhanced upon HIV-1 infection.

Depolarized mitochondria release Cyto c to initiate the mitochondrial apoptotic pathway (45). We therefore examine whether SAMHD1 expression also promotes cytosolic release of Cyto c. Subcellular fractionation was performed in THP-1 Ctrl and SAMHD1 KO cells mock infected or infected with HIV-1-Luc/VSV-G and treated with NVP for 3 dpi to detect cytosolic Cyto c. The cytosolic and mitochondrial fractions of the cell lysate were detected by Western blot. Tubulin was used as the cytosolic marker, and voltage-dependent anion channel (VDAC) was used as the mitochondrial marker. SAMHD1 was detected in both the cytosolic and mitochondrial fractions. Cyto c was more enriched in the cytosolic fraction in THP-1 Ctrl cells than SAMHD1 KO cells, suggesting that SAMHD1 expression enhanced cytosolic release of Cyto c (Fig. 5C and D). HIV-1-Luc/VSV-G infection further promoted cytosolic Cyto c in THP-1 Ctrl cells, which was mitigated by NVP treatment. HIV-1-Luc/VSV-G-induced cytosolic release of Cyto c was alleviated in SAMHD1 KO cells, suggesting that HIV-1-induced cytosolic release of Cyto c depends on SAMHD1 expression. SAMHD1 expression was reconstituted in SAMHD1 KO cells to confirm the role of SAMHD1 in cytosolic release of Cyto C. THP-1 Ctrl, SAMHD1 KO, Lvx, and SAMHD1 KI cell lines were infected with HIV-1-Luc/VSV-G or mock infected for 3 dpi. Consistently, we found that reconstitution of SAMHD1 expression in SAMHD1 KO cells reinforced cytosolic release of Cyto c in the absence or presence of HIV-1-Luc/VSV-G infection to the level comparable to THP-1 Ctrl cells (Fig. 5E and F). Together, these data suggest that SAMHD1 enhances apoptosis induced by HIV-1 infection through the mitochondrial pathway by depolarizing mitochondria and cytosolic release of Cyto c.

### SAMHD1-enhanced apoptosis is associated with increased BIK expression independently of HIV-1 infection

**Fig 6:**
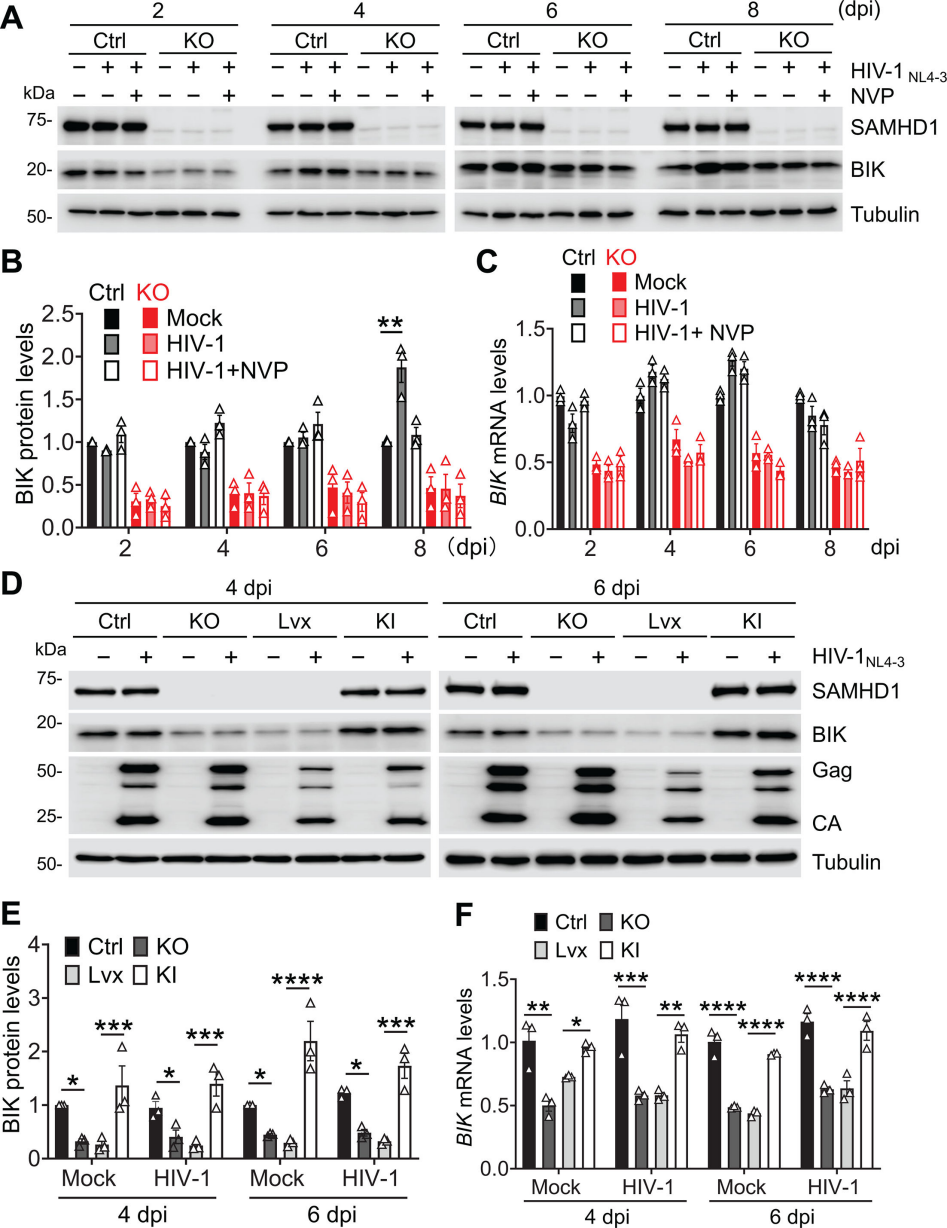
SAMHD1-enhanced apoptosis is associated with increased BIK expression independently of HIV-1 infection. (A, B, and C) THP-1 Ctrl and SAMHD1 KO cell lines were infected with HIV-1_NL4-3_ (MOI = 2) for the indicated time or mock treated. NVP was used to block HIV-1 infection. (D, E, and F) THP-1 Ctrl, SAMHD1 KO, Lvx control, and SAMHD1 KI cell lines were infected with HIV-1_NL4-3_ (MOI = 2) or mock treated for the indicated time. (**A and D**) SAMHD1, BIK, tubulin, and HIV-1 Gag and CA in cells were detected by Western blot. (**B and E**) The relative levels of BIK protein were quantified by densitometry analysis and normalized to tubulin. BIK protein levels of mock-infected THP-1 Ctrl cells were set to 1. (**C and F**) The mRNA levels of BIK were detected by qRT-PCR and were expressed relative to mock-infected THP-1 Ctrl cells that were set to 1. GAPDH was used to normalize qRT-PCR results. (B, E, and F) Unpaired *t*-test (**B**) and one-way ANOVA (**E and F**) were used for statistical significance. *P < 0.05; **P < 0.01; ***P < 0.001. ****P < 0.0001. The data (means ± SEM) represent three independent experiments.

BCL-2 family proteins play an important role in the regulation of the mitochondrial pathway (27, 28). To investigate the mechanism by which SAMHD1 enhances apoptosis induced by HIV-1 infection through the mitochondrial pathway, the expression of BCL-2 family proteins in THP-1 Ctrl and SAMHD1 KO cell lines infected with HIV-1_NL4-3_ and with or without NVP at 2–8 dpi was measured by Western blot. The expression of BIK in THP-1 Ctrl cells, with or without HIV-1 infection, was higher than that in SAMHD1 KO cells at 2, 4, 6, and 8 dpi (Fig. 6A and B; Fig. S7A), while the expressions of BCL-2, BCL-X_L_, BAX, BAK, BIM, and BID were not affected by SAMHD1 expression, HIV-1 infection, or NVP treatment (Fig. S7A), suggesting that SAMHD1-enhanced apoptosis is associated with increased BIK expression. Interestingly, BIK expression was increased by HIV-1 infection at 8 dpi only in THP-1 Ctrl cells but not in SAMHD1 KO cells based on the average of three independent experiments (Fig. 6B).

To investigate whether SAMHD1 affects BIK expression through proteasomal or lysosomal degradation, THP-1 Ctrl and SAMHD1 KO cell lines were treated separately with proteasomal or lysosomal inhibitors 1–2 days before BIK and SAMHD1 detection by Western blot. MG132 is a proteasome inhibitor that induces PARP cleavage (46–48), while Chloroquine (CQ) is a lysosomal hydrolase inhibitor that increases the autophagy marker light chain 3 (LC3) isoform LC3A/B-II accumulation (49, 50). Cleaved PARP and LC3A/B-I/II accumulation were used as positive controls, confirming the validity of the MG132 and CQ treatments. BIK expression was not affected by MG132 or CQ treatment (Fig. S8A), suggesting that SAMHD1 does not regulate BIK expression through the proteasomal or lysosomal degradation pathway.

Our previous study (18) and this study showed that SAMHD1 is localized at the mitochondria and cytosol (Fig. 5C and E). BIK is localized in the endoplasmic reticulum (ER) and translocated to mitochondria when the mitochondrial apoptotic pathway is activated (51). To investigate whether SAMHD1 is associated with BIK, we performed co-immunoprecipitation (Co-IP) in THP-1 Ctrl cells and SAMHD1 KO cells, and we found that SAMHD1 did not interact with BIK in THP-1 cells (Fig. S8B). Then, we further investigated whether HIV-1_NL4-3_ infection affected the interaction between SAMHD1 and BIK in THP-1 Ctrl cells using Co-IP. No interaction between SAMHD1 and BIK was observed in THP-1 cells regardless of HIV-1_NL4-3_ infection (Fig. S8C).

To assess whether BIK expression was affected by SAMHD1 at the transcription level, THP-1 Ctrl and SAMHD1 KO cells were mock infected or infected with HIV-1_NL4-3_ and treated with NVP. At 2–8 dpi, BIK mRNA was measured by qRT-PCR. Overall, the *BIK* mRNA levels were higher in THP-1 Ctrl cells compared to SAMHD1 KO cells, regardless of HIV-1_NL4-3_ infection or NVP treatment (Fig. 6C), suggesting that SAMHD1 increases BIK expression at the mRNA level. *BAX* mRNA, used as a negative control, was not affected by SAMHD1 expression (Fig. S7C).

Next, SAMHD1 was reconstituted in SAMHD1 KO cells to confirm its effect on upregulation of BIK expression. At 4 and 6 dpi, we observed that the levels of BIK protein (Fig. 6D and E) and mRNA (Fig. 6F) in THP-1 Ctrl and SAMHD1 KI cells were higher than those in SAMHD1 KO and Lvx cells, regardless of HIV-1_NL4-3_ infection, suggesting that SAMHD1 increases BIK expression at both the protein and mRNA levels. By contrast, the protein levels of BCL-2, BCl-X_L_, BAX, BAK, BIM, and BID (Fig. S7B), as well as mRNA levels of *BAX* (Fig. S7D), were not affected by SAMHD1 expression or HIV-1_NL4-3_ infection of THP-1 cell lines. These data indicate that SAMHD1-enhanced apoptosis is associated with increased BIK expression, independently of HIV-1 infection.

### Endogenous BIK contributes to SAMHD1-enhanced apoptosis induced by HIV-1 infection

**Fig 7:**
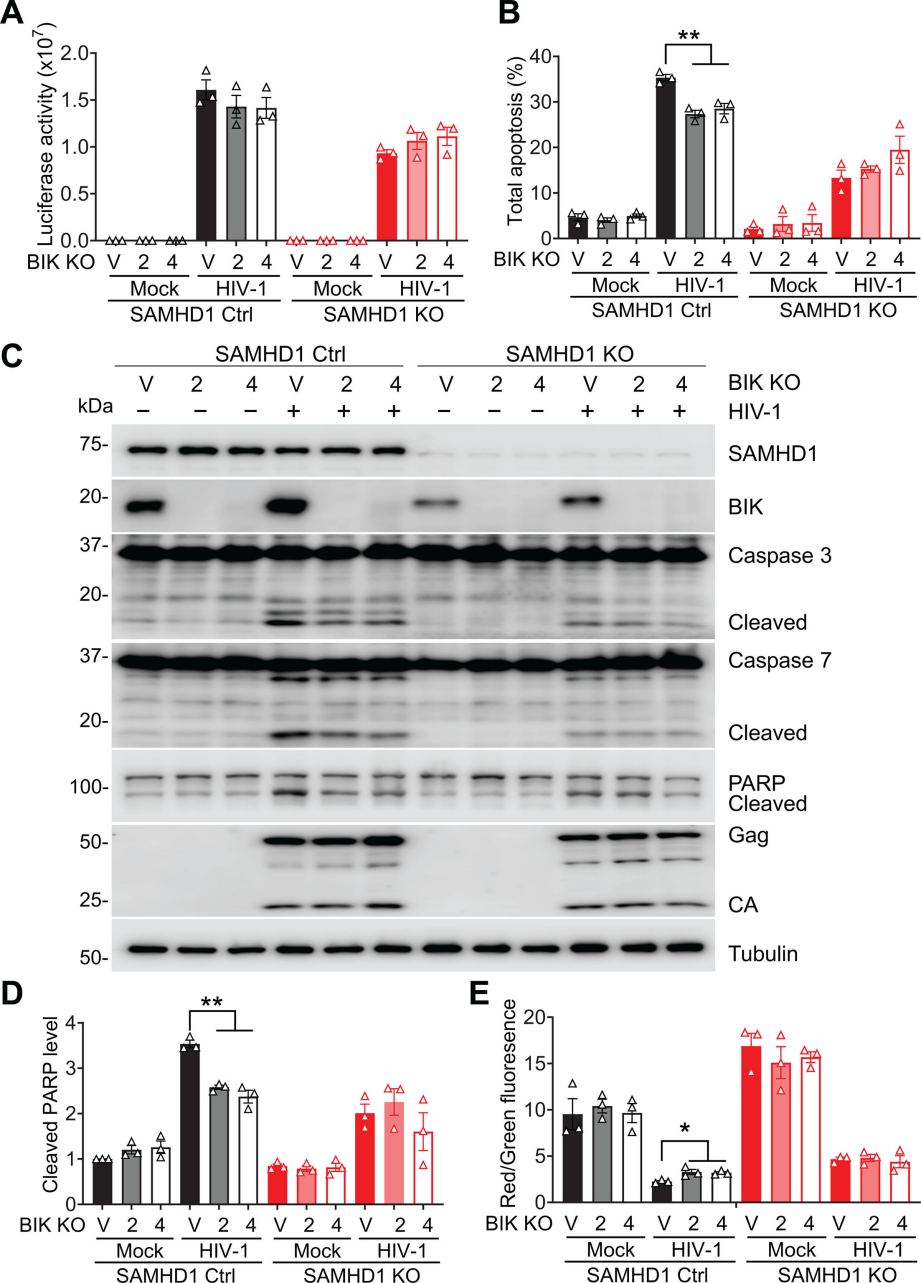
Endogenous BIK contributes to SAMHD1-enhanced apoptosis induced by HIV-1 infection. (**A through E**) THP-1 Ctrl and SAMHD1 KO cell lines expressing empty guide RNA vector (**V**), BIK-specific guide RNA 2 (BIK KO-2), and BIK-specific guide RNA 4 (BIK KO-4) were infected with HIV-1-Luc/VSV-G (MOI = 2) or mock infected. Cells were harvested at 3 dpi for further analysis. (**A**) HIV-1 infection levels were measured by a luciferase assay and normalized to cellular protein concentration. (**B**) Total apoptosis was measured by flow cytometry as indicated in Fig. 1B. Original flow cytometry results are shown in Fig. S9A. (**C**) Detection of SAMHD1, BIK, caspase 3, caspase 7, PARP, HIV-1 Gag and CA, and tubulin by Western blot. Tubulin was used as a loading control. (**D**) The relative cleaved PARP levels were quantified by densitometry analysis and normalized to tubulin. The level of cleaved PARP in mock-infected THP-1 Ctrl V cells was set to 1. (**E**) Cells were stained with JC-1 and measured by flow cytometry. Original flow cytometry results are shown in Fig. S9B. The data in panels A, B, D, and E (means ± SEM) represent three independent experiments. The one-way ANOVA was used for statistical significance. *P < 0.05; **P < 0.01.

To investigate the role of BIK in SAMHD1-enhanced apoptosis, we knocked out BIK expression in THP-1 SAMHD1 Ctrl and SAMHD1 KO cell lines and then measured HIV-1 infection and apoptosis (Fig. 7A and B). BIK knockout was confirmed with Western blot (Fig. 7C). We established six stable THP-1 cell lines with Ctrl empty guide RNA vector (V), Ctrl BIK KO-2 (BIK gRNA-2), Ctrl BIK KO-4 (BIK gRNA-4), SAMHD1 KO V, SAMHD1 and BIK double-KO (DKO)-2 (BIK gRNA-2), and SAMHD1 and BIK DKO-4 (BIK gRNA-4). These cell lines were infected with HIV-1-Luc/VSV-G or mock infected. At 3 dpi, HIV-1-Luc/VSV-G infection in THP-1 cells was not significantly affected when BIK was depleted (Fig. 7A). The total percentages of apoptosis in HIV-1-infected THP-1 Ctrl V cells (35.2%) were significantly higher than those (26.3%–27.6%) observed in Ctrl BIK KO-2 and Ctrl BIK KO-4 cells (Fig. 7B; Fig. S9A), suggesting that BIK depletion decreased HIV-1-induced apoptosis in THP-1 SAMHD1 Ctrl cells. By contrast, BIK depletion had no effect on HIV-1-induced apoptosis in SAMHD1 KO cells (Fig. 7B; Fig. S9A). We further investigated the role of BIK in SAMHD1-enhanced apoptosis by analyzing the cleavage of caspases 3/7 and PARP. BIK depletion reduced HIV-1-induced caspases 3/7 and PARP cleavage in THP-1 Ctrl cells. Comparatively, loss of BIK did not significantly reduce HIV-1-induced caspases 3/7 and PARP cleavage in SAMHD1 KO cell lines (Fig. 7C and D).

BIK is a BH3-only protein that converges apoptotic stimuli, permeabilizes the outer mitochondrial membrane by activating BAX/BAK oligomerization through releasing calcium ions from the ER, and initiates mitochondrial apoptosis (51). We therefore questioned whether BIK was involved in SAMHD1-mediated mitochondrial depolarization. THP-1 Ctrl or SAMHD1 KO cell lines with or without BIK depletion were infected with HIV-1-Luc/VSV-G or mock infected. At 3 dpi, the red/green fluorescence ratio of JC-1 dye staining in THP-1 Ctrl V cells infected with HIV-1 was significantly lower compared to Ctrl BIK KO-2 and Ctrl BIK KO-4 cells, suggesting that BIK depletion replenished Δψm in HIV-1-infected THP-1 Ctrl cells (Fig. 7E; Fig. S9B). Conversely, BIK depletion did not further promote Δψm of HIV-1-infected SAMHD1 KO cells. These data indicate that endogenous BIK mediates SAMHD1-induced mitochondrial depolarization in HIV-1-infected THP-1 cells, suggesting that BIK contributes to SAMHD1-enhanced mitochondrial apoptosis induced by HIV-1 infection.

## DISCUSSION

**Fig 8:**
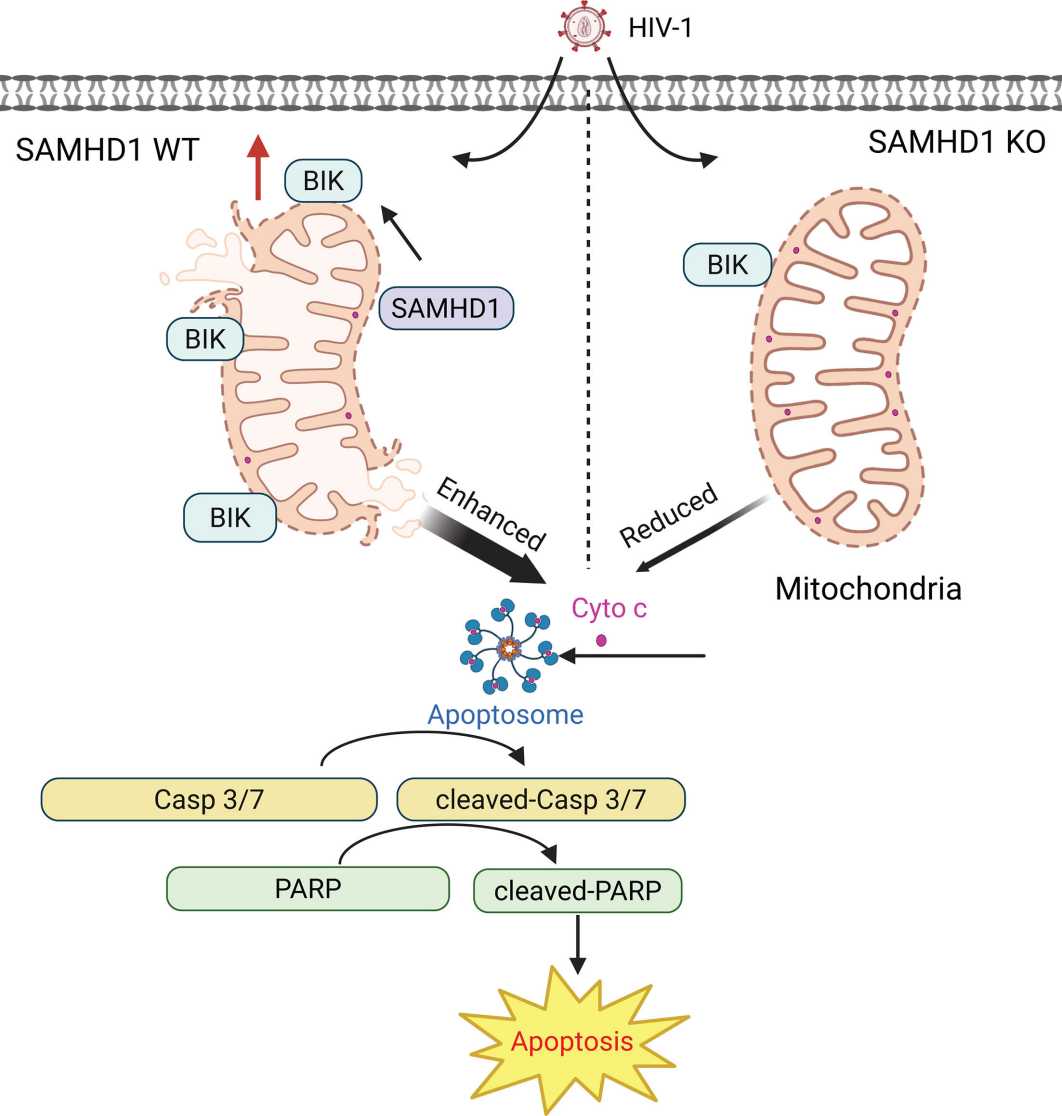
SAMHD1 enhances HIV-1-induced apoptosis in monocytic cells via the mitochondrial pathway. Endogenous SAMHD1 enhances HIV-1-induced apoptosis in undifferentiated monocytic cells. Mechanistically, SAMHD1 increases mitochondrial membrane damage, BIK expression, and Cyto c release into the cytosol of HIV-1-infected cells. As a result, caspases 3/7 are activated by apoptosomes and then cleave downstream PARP, leading to enhanced apoptosis in the presence of SAMHD1. By contrast, SAMHD1 depletion reduces apoptosis in HIV-1-infected monocytic cells. Our findings suggest positive regulatory mechanisms by which SAMHD1 enhances apoptosis induced by HIV-1 infection through the mitochondrial pathway.

Apoptosis is a major form of programmed cell death and plays a crucial role in various physiological processes, including viral infections (27, 28). In this study, we identified that SAMHD1 enhances apoptosis induced by HIV-1 infection in THP-1 cells through the mitochondrial pathway (summarized in Fig. 8). Other studies have shown that SAMHD1 enhances apoptosis in primary human monocytic cells infected with HTLV-1 (39), and overexpression of SAMHD1 in non-small-cell lung cancer A549 cells induces apoptosis (52). Conversely, SAMHD1 knockdown increases apoptosis in ovarian cancer cell lines (53). SAMHD1 silencing also enhances apoptotic cell death in lung adenocarcinoma cells (54). Moreover, SAMHD1 downregulation combined with radiation enhances anti-tumor immune responses to lung adenocarcinoma in mice (54). These studies suggest that SAMHD1-mediated apoptotic cell death may be dependent on the cell types and apoptotic stimuli, which could be used to develop potential therapeutic strategies against cancers.

SAMHD1 did not inhibit HIV-1 infection at 2 and 3 dpi in dividing THP-1 cells (Fig. 1A). In our previous study, SAMHD1 inhibited HIV-1 infection at 1 and 2 dpi in PMA-differentiated THP-1 macrophage-like cells with increased SAMHD1 protein expression (24, 55). Lower dNTP pool in non-dividing macrophage-like cells (higher SAMHD1 expression) does not support HIV-1 reverse transcription, whereas a higher concentration of dNTPs in proliferating monocytic cells (lower SAMHD1 expression) reaches the threshold required for efficient reverse transcription (24, 55). SAMHD1 efficiently inhibits HIV-1 reverse transcription in macrophage-like cells, resulting in a low HIV-1 infection level that hardly induces significant apoptosis (Fig. 4). Therefore, SAMHD1 cannot enhance HIV-1-induced apoptosis in macrophage-like cells. Treatment of NVP partially inhibited HIV-1-induced apoptosis or the reduction of Δψm (Fig. 1 and 5). Single-cycle HIV-1-Luc/VSV-G contains Vpr, a viral protein known to induce apoptosis (37, 38). NVP treatment of cells inhibits HIV-1 reverse transcription but does not affect Vpr incorporated in virions and its expression during infection, which can lead to apoptosis. This is the reason why NVP treatment partially reduces HIV-1-induced apoptosis. Interestingly, we observed that SAMHD1 promoted HIV-1-induced apoptosis in both bystander and HIV-1-infected THP-1 cells (Fig. S3). When added to cultured cells or purified mitochondria, a previous study showed that Vpr protein or Vpr-derived peptides can trigger mitochondrial-dependent apoptosis and mitochondrial depolarization (56). Moreover, Vpr was found to suppress virus-induced innate immune response of both infected and bystander macrophages by targeting PU.1 (57). Whether secreted Vpr or virion-incorporated Vpr contributes to SAMHD1-induced mitochondrial-dependent apoptosis in both infected and bystander cells can be investigated in the future.

In this study, we observed that SAMHD1 enhances HIV-1-induced apoptosis in THP-1 cells by reducing Δψm. A previous study showed that SAMHD1 knockout impairs the Δψm of uninfected mouse macrophages at both the resting state and M1-polarized state (58). Thus, the function of SAMHD1 in maintaining Δψm varies across different cell types and is dependent on apoptotic stimuli. Our previous study showed that SAMHD1 enhances Fas-L-induced apoptosis through the death receptor apoptotic pathway (40). In our study, truncated BID (tBID), which is cleaved from BID by caspase 8 in the death receptor apoptotic pathway (59, 60), was not detected in monocytic THP-1 cells during HIV-1 infection (Fig. S7A and B). This indicates that SAMHD1-enhanced apoptosis in HIV-1-infected monocytic cells does not occur through the death receptor apoptotic pathway. Therefore, SAMHD1 can enhance both the death receptor and mitochondrial apoptotic pathways, depending on the specific stimuli.

In this study, we observed that SAMHD1-enhanced apoptosis is associated with increased BIK expression. BIK is a pro-apoptotic member of the BCL-2 family and binds to anti-apoptotic proteins BCL-2 and BCL-X_L_. The interaction between BIK and BCL-2 or BCL-X_L_ inhibits the function of anti-apoptotic proteins, leading to increased MOMP and Cyto c release (28, 51). A previous study showed that knockdown of BIK in human airway epithelial cells significantly reduced the apoptosis induced by influenza A virus infection, suggesting that BIK is a major mediator for the apoptotic pathway triggered by viral infection (61). Our previous (18) and this study (Fig. 5C and E) showed that SAMHD1 is localized at mitochondria, as confirmed by cytosolic and mitochondrial fractionation. However, we did not detect the interaction between endogenous SAMHD1 and BIK in THP-1 cells. The loss of BIK expression selectively reduces the enhancement of SAMHD1 in HIV-1-induced apoptosis in THP-1 Ctrl cells but does not influence apoptosis levels in SAMHD1 KO cells (Fig. 7). The phenomenon occurs likely because the expression of BIK in THP-1 Ctrl cells is higher than in SAMHD1 KO cells (Fig. 6). Therefore, the loss of higher BIK expression in Ctrl cells leads to a more pronounced reduction in apoptosis compared to SAMHD1 KO cells.

The reduced BIK protein level in SAMHD1 KO cells might have failed the required threshold for BIK protein to trigger apoptosis for HIV-1 infection in THP-1 cells (Fig. 7). Future investigation is required to confirm this possibility. Additional experiments can be performed with reconstitution of BIK expression, the use of BIK-specific apoptotic stimuli, and the use of other cell types and primary monocytes having different levels of SAMHD1 or BIK expression. Alternatively, BIK protein might work with SAMHD1 protein to execute HIV-1-induced apoptosis. Loss of BIK expression reverted Δψm in SAMHD1-expressing THP-1 cells, but not in SAMHD1 KO cells, upon HIV-1 infection, suggesting that BIK is required for SAMHD1-enhanced mitochondrial depolarization by HIV-1 (Fig. 7E; Fig. S9B). HIV-1 infection of SAMHD1-expressing THP-1 cells did not further promote BIK protein expression by 4 dpi, at which SAMHD1-enhanced apoptosis was already observed (Fig. 3A and B and 6A and B). BIK and SAMHD1 interaction in the absence or presence of HIV-1 infection was not detected (Fig. S8C). Therefore, other cellular factors and mechanisms might be involved in SAMHD1/BIK-dependent mitochondrial apoptosis induced by HIV-1 infection. A prior study showed that SAMHD1 interacts with the mitochondrial protein VDAC1 in mouse macrophages (58). We have reported that SAMHD1 interacts with the mitochondrial antiviral-signaling protein in THP-1 cells (18). It is also possible that SAMHD1 interacts with other mitochondrial proteins that contribute to enhanced mitochondrial apoptosis.

The dNTP hydrolase (dNTPase) activity of SAMHD1 is critical for its HIV-1 restriction function in non-dividing immune cells (4, 6, 55, 62–64). We have reported that the dNTPase activity of SAMHD1 is important for its suppression of innate immune responses in differentiated monocytic cells, but not in dividing HEK293T cells (17, 18, 43). Further study is required to investigate whether the dNTPase activity of SAMHD1 is important for its function in enhancing HIV-1-induced mitochondrial apoptosis. In this type of future study, it is challenging but necessary to distinguish SAMHD1-mediated HIV-1 restriction from the effects on virus-induced apoptosis in cells.

We observed that SAMHD1 enhanced the MG-132-induced apoptosis in THP-1 cells (Fig. S8A), suggesting that SAMHD1 plays an important role in regulating cell death. An *in vivo* study using humanized mice and nonhuman primates reported that treatment of lung CD4+ T cells with the pan-caspase inhibitor Z-VAD-FMK and caspase 3 inhibitor Z-DEVD-FMK decreased cell death induced by HIV-1 and SIV infection, suggesting that HIV-1-induced apoptosis significantly contributes to the loss of CD4+ T cells (65). Further studies are needed to investigate the role of SAMHD1 in HIV-1-induced loss of CD4+ T cells.

Apoptosis plays a crucial role in HIV-1 pathogenesis. Multiple HIV-1 proteins modulate the activation of apoptosis, which not only assists HIV-1 infection by escaping host immune surveillance but also causes a significant decline in immune cell count, especially CD4+ T cells (36, 65, 66). The underlying mechanisms are not clear. Macrophages and CD4+ T cells are the primary target cells of HIV-1 infection, depending on HIV-1 subtypes, while monocytes are not primary target cells for HIV-1 as they are resistant to productive HIV-1 infection (67–69). The *in vivo* function of HIV-1-induced apoptosis in monocyte cells, macrophage cells, and CD4+ T cells remains to be explored. Particularly, we observed that SAMHD1 has dual functions: (i) restricting HIV-1 replication and (ii) promoting HIV-1-induced apoptosis, which implicates both infected and bystander cells (Fig. S3). In monocytic cells, SAMHD1 was not effective in restricting HIV-1 replication but promoted HIV-1-induced apoptosis (Fig. 1 to 4A and B) through the BIK-dependent mitochondrial pathway (Fig. 5 to 7). In stark contrast, SAMHD1 was restrictive to HIV-1 replication in terminally differentiated macrophage-like cells (Fig. 4C and D). The repressed HIV-1 replication also decreased HIV-1-induced apoptosis in SAMHD1-expressing macrophage-like cells. Therefore, the SAMHD1-enhanced HIV-1-induced apoptosis requires efficient HIV-1 replication. Whether SAMHD1 promotes HIV-1-induced apoptosis to macrophages *in vivo* or in the co-cultured model would be worth investigating. It is possible that SAMHD1-expressing macrophages undergo enhanced HIV-1-induced apoptosis as bystander cells when co-cultured with HIV-1-infected permissive cells (Fig. S3). CD4+ T cells are proficient SAMHD1-expressing cells (6, 70). It will be of interest to investigate whether SAMHD1 also contributes to HIV-1-induced apoptosis of CD4+ T cells (activated or resting CD4+ T cells), as infected cells or bystander cells.

In summary, we demonstrated that endogenous SAMHD1 enhances apoptosis induced by HIV-1 infection through the mitochondrial pathway in monocytic cells. Our results provide new insights into the mechanisms underlying the apoptotic function of SAMHD1 during HIV-1 infection.

## MATERIALS AND METHODS

### Cell lines

THP-1 Ctrl, SAMHD1 KO, Lvx, and SAMHD1 KI cell lines were described (17, 24). Clones 1 and 2 of THP-1 Ctrl and SAMHD1 KO were from the same treatment but isolated as independent single colonies of the treated populations. U937 EV and U937 SAMHD1 cell lines were described (43). HEK293T cells and GHOST/R5/X4 cells were described (17). HEK293T cells were cultured in DMEM with 10% FBS, 100 U/mL penicillin, and 100 µg/mL streptomycin. GHOST/R5/X4 were cultured in DMEM with 10% FBS, 100 U/mL penicillin, 100 µg/mL streptomycin, 500 µg/mL neomycin, 50 µg/mL hygromycin B, and 1 µg/mL puromycin. THP-1 BIK KO cell lines were generated by CRISPR/Cas9 technology and grown in RPMI 1640 media with 10% fetal bovine serum (FBS), 100 U/mL penicillin, 100 µg/mL streptomycin, 1 µg/mL puromycin, and 10 µg/mL blasticidin.

### CRISPR/Cas9-mediated gene knockout

HEK293T cells were transfected with lentiCRISPR v2-Blast, or lentiCRISPR v2-Blast-BIK gRNA, psPAX2, and pMD2.G (ratio 4:3:1) by using jetPRIME transfection reagent. Two Guide RNA-target BIK sequences were 5′-TATGGAGGACTTCGATTCTT-3′ and 5′ CCTGGAACCCCCGACCATGG-3′. After 48 h transfection, the lentivirus was collected and purified by passing it through a 0.45 µm filter. THP-1 ctrl and SAMHD1 KO cells were transduced with lentivirus in the presence of 10 µg/mL polybrene. At 48 h post-transduction, the cells were cultured in RPMI 1640 media with blasticidin (10 µg/mL). The BIK knockout cells were confirmed by Western blot.

### Virus stocks and viral infection assays

Single-cycle HIV-1-Luc and HIV-1-GFP pseudotyped with vesicular stomatitis virus G protein (VSV-G) and replication-competent HIV-1_NL4-3_ were generated from HEK293T cells. Virus stock was digested with DNase I (40 U/mL) for 1 h at 37°C and filtered (0.45 µm filter). The infectious units of HIV-1 were measured at limiting dilution on GHOST/X4/R5 cells as described (55, 71). THP-1 and U937 cell lines were infected with HIV-1-Luc/VSV-G or HIV-1-GFP/VSV-G at a multiplicity of infection (MOI) of 2 in the presence of 10 µg/mL polybrene. The infection level of HIV-1 was determined by luciferase assay according to the manufacturer’s instructions. Luciferase values were normalized by protein concentration. PMA-differentiated THP-1 and U937 cells were infected with HIV-1-Luc/VSV-G (MOI = 2) in the presence of 10 µg/mL polybrene by spinoculation at 2,000 × *g* for 2 h at room temperature. THP-1 cells were infected with HIV-1_NL4-3_ (MOI = 2) in the presence of 10 µg/mL polybrene by spinoculation at 2,000 × *g* for 2 h at room temperature.

### Western blot

Western blot analysis was performed as previously described (19). Briefly, cells were lysed in the cell lysis buffer with the protease inhibitor cocktail and phosphatase inhibitor cocktail. The concentration of protein was measured by the bicinchoninic acid (BCA) assay. The same amounts of protein were separated by SDS-PAGE and transferred onto the membrane. The membranes were blocked in 5% non-fat milk for 1 h and incubated with the indicated primary antibody overnight. The membranes were incubated with HPR-labeled secondary antibody for 1 h and developed by Odyssey Fc Imager. Tubulin was used as a loading control to normalize quantification by densitometry.

### Cytosolic and mitochondrial fractionation

THP-1 cells were infected with HIV-1-Luc/VSV-G or mock infected for 3 dpi. The cytosolic and mitochondrial fractions of THP-1 cells were separated using the Cell Fractionation Kit (Abcam) according to the manufacturer’s instructions. Protease inhibitor cocktail and phosphatase inhibitor cocktail were used in the cell lysis buffer. Cytosolic and mitochondrial proteins were analyzed by Western blot. VDAC and tubulin were used as mitochondrial and cytosolic markers (18), respectively.

### Treatment of cells with NVP, F-DEVD-FMK, PMA, MG132, or chloroquine

THP-1 cells and U937 cells were infected with HIV-1-Luc/VSV-G, HIV-1-GFP/VSV-G, or HIV-1_NL4-3_. NVP (10 mM) or F-DEVD-FMK (20 µM) was maintained in the medium throughout the infection and subsequent culture. THP-1 and U937 cell lines were treated with PMA (30 ng/mL) for 1 day and then cultured with fresh medium for 1 day. THP-1 cells were incubated with MG132 (1 µM) and chloroquine (100 µM) for 3 h and then cultured with fresh medium. After 1 and 2 days of treatment, cells were harvested for Western blot.

### RNA extraction and qRT-PCR

THP-1 cells were collected after HIV-1 infection for the indicated time. Total RNA was extracted using RNeasy Plus Kits for RNA Isolation according to the manufacturer’s instructions. Total RNA (1 µg per sample) was reverse transcribed using the iScript cDNA Synthesis Kit. iTaq Universal SYBR Green supermix was used for qPCR. GAPDH mRNA was used as the housekeeping gene to normalize the expression of the target gene (18).

### Caspase 3/7 activity assay

Caspase 3/7 activity assay was performed as previously described (24). Briefly, THP-1 cells (1 × 10^6^ per well) were seeded in the six-well plates. After the HIV-1 infection for the indicated time, cells (2 × 10^4^) were transferred into 96-well plates (in six replicates). Caspase 3/7 activity was measured by Caspase-Glo 3/7 Assay System according to the manufacturer’s instructions. Briefly, 100 µL of Caspase-Glo 3/7 Substrate was added to 96-well plates, and the plates were incubated for 1 h at room temperature. The luciferase activity was measured by the microplate reader (VICTOR Nivo).

### Co-immunoprecipitation (IP) assay

THP-1 cells were harvested and lysed in the cell lysis buffer. BIK antibody (2 µg per 1 × 10^7^ cells) or SAMHD1 antibody (2 µg per 1 × 10^7^ cells) and Dynabeads Protein G magnetic beads were used for IP. The same amounts of nonspecific rabbit or mouse IgG were used as a negative control. The bound beads were washed with PBS with 0.1% Tween 20 three times and boiled in protein loading buffer. The input and IP products were detected by Western blot.

### Statistical analysis

All results were shown as mean ± SEM. GraphPad Prism software was used to analyze data. The unpaired *t*-test and one-way ANOVA were used for analysis, and statistical significance was defined as *P* < 0.05.

## Data Availability

All data and material availability are included in the paper and supplemental information.

## SUPPLEMENTAL MATERIAL

Supplemental figures mbio.00425-25-s0001.pdf Fig. S1-S9.

Table S1 mbio.00425-25-s0002.pdf Key reagents and resources.

ASM does not own the copyrights to Supplemental Material that may be linked to, or accessed through, an article. The authors have granted ASM a non-exclusive, world-wide license to publish the Supplemental Material files. Please contact the corresponding author directly for reuse.
