# Supplementary material for: SAMHD1 enhances HIV-1-induced apoptosis in monocytic cells via the mitochondrial pathway
Source: mBio. 2025 May 28;16(7):e00425-25. doi: 10.1128/mbio.00425-25 (PMC12239581; doi:10.1128/mbio.00425-25)
Supplement: Table S1 — Key reagents and resources. [file mbio.00425-25-s0002.pdf]

TABLE S1: KEY REAGENTS AND RESOURCES

REAGENT or RESOURCE	SOURCE	IDENTIFIER
Antibodies		
Mouse monoclonal anti-SAMHD1	Abcam	OTI1F9
Rabbit polyclonal anti-caspase 3	Cell Signaling Technologies (CST)	9662
Rabbit polyclonal anti-caspase 7	CST	9492
Rabbit polyclonal anti-PARP	CST	9542
Mouse monoclonal anti-HIV-1 p24 (capsid)	The NIH AIDS Reagent Program	ARP-4121
Mouse monoclonal anti-tubulin	Abcam	Ab7291
Rabbit monoclonal anti-cytochrome c	CST	11940
Rabbit monoclonal anti-VDAC	CST	4661
Rabbit polyclonal anti-BIK	CST	4592
Rabbit monoclonal anti-BCL-2	CST	4223
Rabbit monoclonal anti-BCL-X_L	CST	2764
Rabbit monoclonal anti-BAX	CST	5023
Rabbit monoclonal anti-BAK	CST	12105
Rabbit monoclonal anti-BIM	CST	2933
Rabbit polyclonal anti-BID	CST	2002
Rabbit monoclonal anti-LC3A/B	CST	12741
Goat anti-mouse IgG (H+L) HRP	Promega	W4021
Goat anti-rabbit IgG (H+L) HRP	Promega	W401B
Mouse IgG isotype control	Invitrogen	02-6502
Rabbit anti-mouse IgG antibody	MilliporeSigma	AP160
Bacterial and virus strains		
Single-cycle, VSV-G-pseudotyped luciferase reporter HIV-1 (based on pNL4-3E-R+)	The NIH AIDS Reagent Program (1)	Generated from pNL4-3E-R+ luciferase
Single-cycle, VSV-G-pseudotyped GFP reporter HIV-1 (based on pNL4-3E-R+)	The NIH AIDS Reagent Program (2)	Generated from pNL4-3E-R+ GFP
HIV-1_{NL4-3}	The NIH AIDS Reagent Program (3)	Generated from pNL4-3
Chemicals, peptides, and recombinant proteins		
DMEM	Gibco	11965-092
RPMI-1640 Medium	ATCC	30-2001
Fetal Bovine Serum (FBS)	R&D Systems	S1150H
Penicillin and Streptomycin	Gibco	15140-122
OPTI-MEM serum-free media	Gibco	31985-062
Polyethyleneimine (PEI)	Polysciences	24313-2
Puromycin	MilliporeSigma	P8833
Nevirapine (NVP)	The NIH AIDS Reagent Program	4666
Phorbol 12-myristate 13-acetate (PMA)	MilliporeSigma	P8139
Z-DEVD-FMK	MCE	HY-12466
JC-1	CST	92891

Cell lysis buffer	CST	9803
Protease inhibitor cocktail	MilliporeSigma	P8340
Phosphatase inhibitor cocktail	MilliporeSigma	P0044
Blasticidin S HCl	Gibco	A1113903
jetPRIME® transfection reagent	Polyplus	101000046
Geneticin (G418)	Gibco	10131035
MG132	Santa Cruz	sc-201270
Chloroquine	MilliporeSigma	C6628
Dynabeads protein G	Invitrogen	10004D
Critical commercial assays		
Luciferase Assay System	Promega	E1500
BD Pharmingen™ FITC Annexin V Apoptosis detection Kit I	BD Biosciences	556547
Caspase-Glo® 3/7 Assay System	Promega	G8093
Cell Fractionation Kit - Standard	Abcam	ab109719
RNeasy Plus Kits for RNA Isolation	Qiagen	74134
iScript™ cDNA Synthesis Kit	BIO-RAD	1708891
iTaq™ Universal SYBR® Green Supermix	BIO-RAD	1725124
Pierce™ BCA Protein Assay Kits	Thermo Fisher Scientific	23225
SuperSignal West Femto Maximum Sensitivity Substrate	Thermo Fisher Scientific	34094
Experimental models: Cell lines		
THP-1 vector control (ctrl), SAMHD1 knockout (KO), Lvx vector control and SAMHD1 knockin (KI)	The Wu lab (1)	N/A
U937 Empty Vector (EV) and SAMHD1 expression	The Wu lab (4)	N/A
GHOST/R5/X4 cell line	Vineet KewalRamani (5)	N/A
HEK293T cell line	ATCC	CRL-3216
THP-1 ctrl empty guide RNA vectors (V), ctrl BIK-KO, SAMHD1 KO V and SAMHD1 and BIK- double KO(DKO)	This study	N/A
Oligonucleotides (F, forward; R, reverse)		
BIK for qRT-PCR, F 5-ATCTTGATGGAGACCCTCCTGT-3’	IDT	Ref. (6)
BIK for qRT-PCR, R 5’-CACTGCCCTCCATGCATT-3’	IDT	Ref. (6)
BAX for qRT-PCR, F 5’-CCCGAGAGGTCTTTTTCCGAG-3’	IDT	Ref. (7)
BAX for qRT-PCR, R 5’-CCAGCCCATGATGGTTCTGAT-3’	IDT	Ref. (7)
GAPDH for qRT-PCR, F 5'-GGAAGGTGAAGGTCGGAGTCAACGG-3'	IDT	N/A
GAPDH for qRT-PCR, R 5'-CTGTTGTCATACTTCTCATGGTTCAC-3'	IDT	N/A
BIK gRNA-2, F 5’-CACCGAAGAATCGAAGTCCTCCATA-3’	IDT	N/A
BIK gRNA-2, R 5’-AAACTATGGAGGACTTCGATTCTTC-3	IDT	N/A
BIK gRNA-4, F 5’-CACCGCCTGGAACCCCCGACCATGG-3’	IDT	N/A

BIK gRNA-4, R 5' AAACCCATGGTCGGGGGTTCCAGGC-3'	IDT	N/A
Recombinant DNA		
pNL4-3E-R+ Luciferase	The NIH AIDS Reagent Program (8)	N/A
pNL4-3E-R+ GFP	The NIH AIDS Reagent Program (2)	
pNL4-3	The NIH AIDS Reagent Program	ARP-114
lentiCRISPR v2-Blast	The Wu lab (9)	N/A
lentiCRISPR v2-Blast-BIK	This study	N/A
pMD2.G	Addgene	12259
psPAX2	Addgene	12260
Software and algorithms		
Flowjo V10	BD Biosciences	Home | FlowJo, LLC
GraphPad Prism 10	GraphPad	Prism - GraphPad

N/A, not available.

References for key reagents and resources:

1. Bonifati S, Daly MB, St Gelais C, Kim SH, Hollenbaugh JA, Shepard C, Kennedy EM, Kim DH, Schinazi RF, Kim B, Wu L. 2016. SAMHD1 controls cell cycle status, apoptosis and HIV-1 infection in monocytic THP-1 cells. Virology 495:92-100.
2. Antonucci Jenna M, Kim Sun H, St. Gelais C, Bonifati S, Li T-W, Buzovetsky O, Knecht Kirsten M, Duchon Alice A, Xiong Y, Musier-Forsyth K, Wu L. 2018. SAMHD1 Impairs HIV-1 Gene Expression and Negatively Modulates Reactivation of Viral Latency in CD4+ T Cells. Journal of Virology 92:10.1128/jvi.00292-18.
3. Phillips S, Baek A, Kim S, Chen S, Wu L. 2022. Protocol for the generation of HIV-1 genomic RNA with altered levels of N (6)-methyladenosine. STAR Protoc 3:101616.
4. Qin Z, Bonifati S, St Gelais C, Li TW, Kim SH, Antonucci JM, Mahboubi B, Yount JS, Xiong Y, Kim B, Wu L. 2020. The dNTPase activity of SAMHD1 is important for its suppression of innate immune responses in differentiated monocytic cells. J Biol Chem 295:1575-1586.
5. St Gelais C, de Silva S, Amie SM, Coleman CM, Hoy H, Hollenbaugh JA, Kim B, Wu L. 2012. SAMHD1 restricts HIV-1 infection in dendritic cells (DCs) by dNTP depletion, but its expression in DCs and primary CD4+ T-lymphocytes cannot be upregulated by interferons. Retrovirology 9:105.
6. Bolden JE, Shi W, Jankowski K, Kan CY, Cluse L, Martin BP, MacKenzie KL, Smyth GK, Johnstone RW. 2013. HDAC inhibitors induce tumor-cell-selective pro-apoptotic transcriptional responses. Cell Death Dis 4:e519.
7. Shen J, Yang H, Qiao X, Chen Y, Zheng L, Lin J, Lang J, Yu Q, Wang Z. 2023. The E3 ubiquitin ligase TRIM17 promotes gastric cancer survival and progression via controlling BAX stability and antagonizing apoptosis. Cell Death Differ 30:2322-2335.

8. Chen S, Bonifati S, Qin Z, St Gelais C, Kodigepalli KM, Barrett BS, Kim SH, Antonucci JM, Ladner KJ, Buzovetsky O, Knecht KM, Xiong Y, Yount JS, Guttridge DC, Santiago ML, Wu L. 2018. SAMHD1 suppresses innate immune responses to viral infections and inflammatory stimuli by inhibiting the NF-kappaB and interferon pathways. Proc Natl Acad Sci U S A 115:E3798-E3807.
9. Espada CE, Sari L, Cahill MP, Yang H, Phillips S, Martinez N, Kenney AD, Yount JS, Xiong Y, Lin MM, Wu L. 2023. SAMHD1 impairs type I interferon induction through the MAVS, IKKepsilon, and IRF7 signaling axis during viral infection. J Biol Chem 299:104925.
